# RNAseq expression analysis of resistant and susceptible mice after influenza A virus infection identifies novel genes associated with virus replication and important for host resistance to infection

**DOI:** 10.1186/s12864-015-1867-8

**Published:** 2015-09-02

**Authors:** Esther Wilk, Ashutosh K. Pandey, Sarah Rebecca Leist, Bastian Hatesuer, Matthias Preusse, Claudia Pommerenke, Junxi Wang, Klaus Schughart

**Affiliations:** Department of Infection Genetics, Helmholtz Centre for Infection Research and University of Veterinary Medicine Hannover, Inhoffenstr. 7, 38124 Braunschweig, Germany; Department of Genetics, Genomics and Informatics, Center for Integrative and Translational Genomics, University of Tennessee Health Science Center, 855 Madison Avenue, Memphis, TN 38163 USA; Institute for Experimental Infection Research, TWINCORE Centre for Experimental and Clinical Infection Research, Hannover, Germany; Department of Molecular Bacteriology, Helmholtz Centre for Infection Research, Braunschweig, Germany; Leibniz-Institut DSMZ-Deutsche Sammlung von Mikroorganismen und Zellkulturen GmbH, Inhoffenstr. 7B, 38124 Braunschweig, Germany; Bioinformatics and Statistics, Helmholtz Centre for Infection Research, Braunschweig, Germany; Department of Microbiology, Immunology and Biochemistry, University of Tennessee Health Science Center, Memphis, USA

## Abstract

**Background:**

The host response to influenza A infections is strongly influenced by host genetic factors. Animal models of genetically diverse mouse strains are well suited to identify host genes involved in severe pathology, viral replication and immune responses. Here, we have utilized a dual RNAseq approach that allowed us to investigate both viral and host gene expression in the same individual mouse after H1N1 infection.

**Results:**

We performed a detailed expression analysis to identify (i) correlations between changes in expression of host and virus genes, (ii) host genes involved in viral replication, and (iii) genes showing differential expression between two mouse strains that strongly differ in resistance to influenza infections. These genes may be key players involved in regulating the differences in pathogenesis and host defense mechanisms after influenza A infections. Expression levels of influenza segments correlated well with the viral load and may thus be used as surrogates for conventional viral load measurements. Furthermore, we investigated the functional role of two genes, *Reg3g* and *Irf7*, in knock-out mice and found that deletion of the *Irf7* gene renders the host highly susceptible to H1N1 infection.

**Conclusions:**

Using RNAseq analysis we identified novel genes important for viral replication or the host defense. This study adds further important knowledge to host-pathogen-interactions and suggests additional candidates that are crucial for host susceptibility or survival during influenza A infections.

**Electronic supplementary material:**

The online version of this article (doi:10.1186/s12864-015-1867-8) contains supplementary material, which is available to authorized users.

## Background

Influenza A viruses have an adverse impact on human and animal health worldwide through seasonal epidemics, newly emerging pandemics, and reoccurring outbreaks in livestock. The most severe human pandemic in 1918 resulted in about 30 million fatal casualties [1]. In addition, seasonal influenza infections represent a major health hazard causing deaths and enormous losses of work force every year [2].

We and others have shown in animal models that the genetic background of the host strongly influences mortality and morbidity after influenza infections. In particular, major differences in susceptibility and resistance were observed between different mouse inbred strains [3–13]. Detailed analysis of the mouse strains C57BL/6J and DBA/2J revealed that C57BL/6J mice survived infections with a low pathogenic A/Puerto Rico/8/1934 H1N1 virus (PR8M) whereas DBA/2J mice rapidly lost weight and all infected mice died [3, 14]. Infected DBA/2J had higher viral loads in their lungs and also exhibited a stronger inflammatory response compared to C57BL/6J mice [3, 14, 15]. Therefore, the comparison of these two mouse strains represents a very suitable model system to identify genes that are associated with severe infection outcomes in humans [16].

During an acute influenza virus infection, highly dynamic and inter-related responses are triggered in the host which eventually results in clearance of the pathogen and establishment of a long-lasting immunity. We recently demonstrated that these host responses can be studied comprehensively by measuring changes in the gene expression levels after infection [17, 18].

Here, we expanded those earlier studies by utilizing a dual RNAseq approach that enabled us to investigate both virus as well as host gene expression in the same individual. We found several new host genes that are strongly correlated with virus gene expression. Host genes potentially involved in viral replication were identified by comparisons with candidates from previous siRNA studies. In addition, we identified host genes that exhibit differential expression between the C57BL/6J and DBA/2J mouse strains after infection. These genes may be crucial to direct the host response to influenza A infections and be causal for differences in susceptibility and resistance of genetically diverse hosts to influenza or other viral infections. We studied the role of two candidate genes and found that deletion *Irf7* renders the host highly susceptible to H1N1 infection.

## Results

### Global expression profiles are distinct in C57BL/6J and DBA/2J mice

**Fig. 1:**
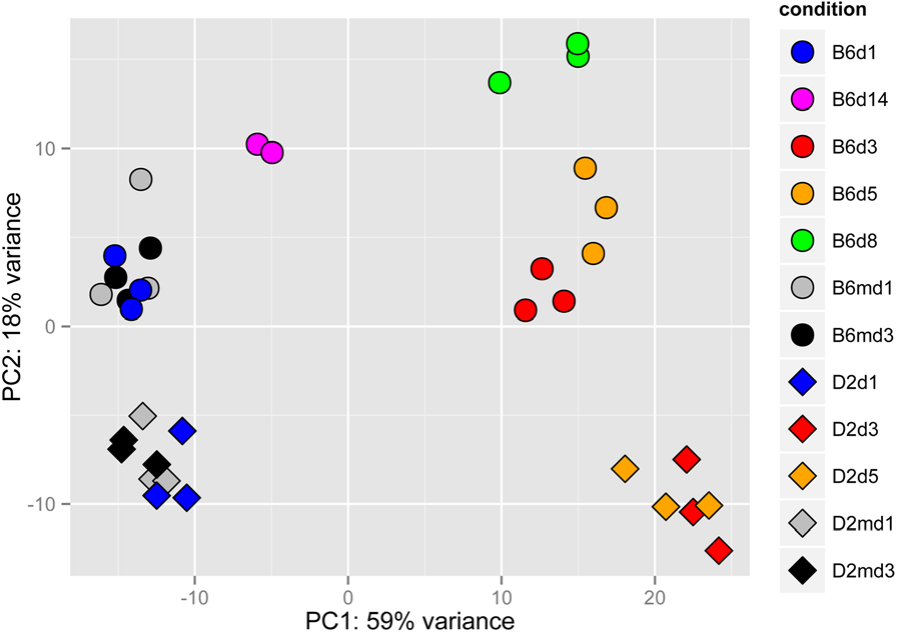
PCA analysis of normalized host gene counts for all samples. Principle component analysis reveals separate grouping of non-infected mice and infected mice for both mouse strains. Replicates for a given day p.i. grouped together well. For C57BL/6J mice, groups from different days p.i. were well separated. For DBA/2J a much stronger infection response was observed compared to C57BL/6J mice and individual mice at days 3 and 5 p.i. were not well separated. Note that for day 14 p.i., two of three samples were not separated and only two spots are visible. B6md1, D2md1 and B6md3, D2md3: C57BL/6J and DBA/2J mice mock-treated and analyzed at days 1 or 3 post treatment, respectively. Sample labels: C57BL/6J at days 1, 3, 5, 8 and 14 p.i.: B6d1, B6d3, B6d5, B6d8, B6d14, respectively; and DBA/2J mice at days 1, 3, 5 p.i.: D2d1, D2d3, D2d5, respectively

RNA was extracted from the lungs of C57BL/6J and DBA/2J mice infected with PR8M (a variant of A/Puerto Rico/8/1934 H1N1) as described in [14], and gene expression was quantified using RNA sequencing (RNAseq) technology. Principal component analysis (PCA) of normalized counts for host genes confirmed separate groupings of non-infected (controls) and infected lungs (Fig. 1). The transcriptome profiles of C57BL/6J mice and DBA/2J mice were distinct as shown by the second principle component, whereas the host response to the infection is mostly represented by the first principle component which explains 59 % of the expression variation. PC2 reveals distinct expression profiles for the two strains due to their different genetic backgrounds explaining 18 % of the expression variation. C57BL/6J mice exhibited a change in transcriptome profiles that was distinct for days 3, 5, 8, and 14 after infection. However, infected DBA/2J mice showed an early and stronger change in transcriptome profiles at day 3 post infection (p.i.) compared to C57BL/6J. Their expression profile did not show any major changes until day 5 when DBA/2J mice were moribund.

### Expression levels of influenza gene segments correlate with viral load

**Fig. 2:**
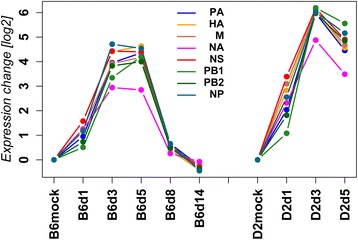
Expression levels of influenza genes. Normalized expression levels for influenza segments (PA, HA, M, NA, NS, PB1, PB2, NP) were calculated as mean expression values (log_2_ RPKM + 1), relative to respective mock treated animals (mock day 1 for day1 infected mice; mock day 3 for all other days p.i.). Lines represent expression levels from lungs of C57BL/6J at day 1, 3, 5, 8 and 14 p.i. (B6d1, B6d3, B6d5, B6d8, B6d14, respectively) and at day 1, 3, 5 p.i. for DBA/2J mice (D2d1, D2d3, D2d5, respectively). B6mock, D2mock: mock-treated C57BL/6J and DBA/2J control mice, respectively

In addition to expression profiling of host genes, RNAseq also allowed us to investigate transcripts of the eight viral segments. Expression levels of all influenza segments (calculated as RPKM: reads which map per kilobase of exon model per million mapped reads) changed in all infected mice over time and were highest at days 3 and 5 p.i. in C57BL/6J mice and at day 3 p.i. in DBA/2J mice (Fig. 2). In infected C57BL/6J mice, expression signals from influenza genes strongly decreased on day 8 p.i. and were at baseline levels of mock-treated controls on day 14 p.i. Expression levels of influenza transcripts were higher in DBA/2J mice compared to C57BL/6J mice at days 1 and 3 p.i. Most influenza RNA segments revealed a similar relative increase in expression, except for the segment encoding the neuraminidase (‘NA’) showing a lower increase compared to all other segments.

**Fig. 3:**
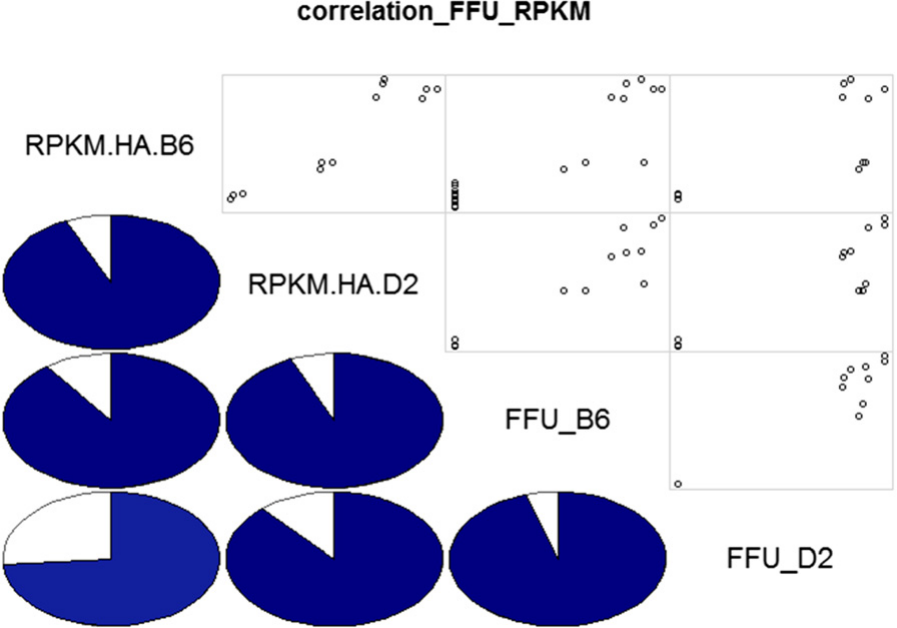
Correlation of expression levels and infectious particles. The part below the diagonal represents Spearman pairwise correlation factors in percent as pie charts; the part above the diagonal shows scatter plots for pairwise comparisons of RPKM and FFU in C57BL/6J and DBA/2J mice. For C57BL/6J mice, RPKM values from mock day3 and from days 1 to 14 p.i., and for DBA/2J, RPKM values from mock day 3 and days 1 to 5 p.i. were used for the analysis. Data were ordered by day, +1 added and then log_2_ transformed. FFU were taken from [15], offset by 1 and log_2_ transformed. Pearson pairwise correlations between RPKM and FFU for C57BL/6J were: corr = 0.8999419, *p*-value = 3.676e-07; and for DBA/2J: cor = 0.8832289, *p*-value = 0.0001401

Dynamics of the influenza gene expression levels determined by RNASeq correlated well to infectious viral particles [15] in C57BL/6J and DBA/2J mice (Fig. 3).

### Differentially expressed genes overlap with genes previously identified to be required for viral replication

**Fig. 4:**
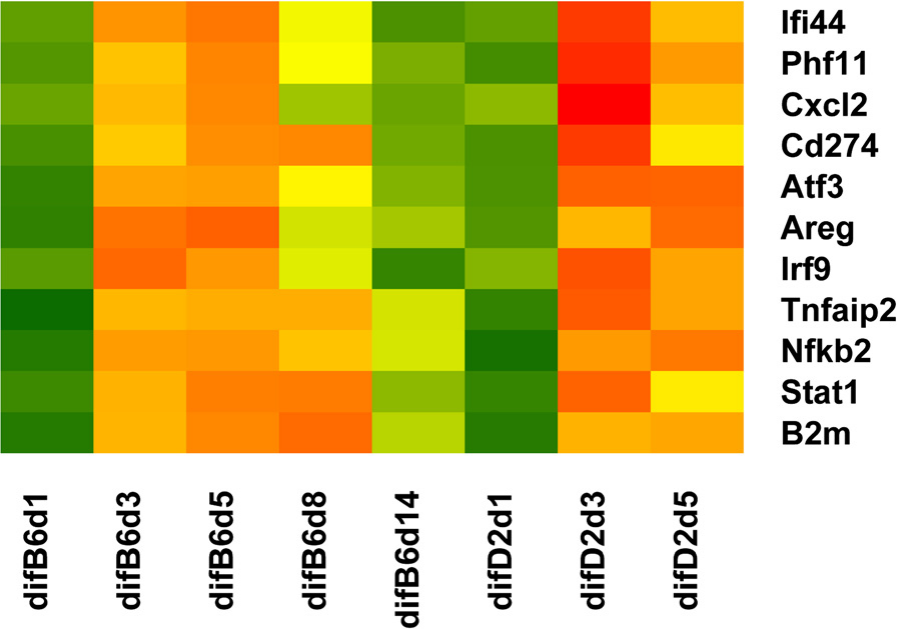
Expression changes of DEG genes overlapping with previously identified genes required for viral replication. The heatmap illustrates DEG genes from infected C57BL/6J and DBA/2J that overlap with previously identified genes [19]. Mean expression differences of DEG genes from infected C57BL/6J and DBA/2J at days 3 and 5 p.i. to mock-treated samples were calculated and values were scaled by rows. Colors display z-scores from −1.5 (dark green) to 1.5 (red) for normalized gene expression values. Rows: name of genes, columns: difB6d1: difference in expression levels of C57BL/6J infected mice at day 1 compared to mock day 1 treated animals; difB6d3 to difB6d14: difference in expression levels of C57BL/6J infected mice at days 3, 5, 8, 15 p.i. compared to mock-treated day 3 animals; difD2d1: difference in expression levels of DBA/2J infected mice at day 1 compared to mock-treated day 1 animals; difD2d3 to difD2d5: difference in expression levels of DBA/2J mice infected at days 3, 5 p.i. compared to mock-treated day 3 animals

Differentially expressed genes (DEG) between infected and mock-treated animals (log-fold change > |0.5|, FDR < 5 %) were determined for C57BL/6J infected mice at days 3, 5 and 8 p.i and for DBA/2J infected mice at days 3 and 5 p.i. (Table 1). We then compared these DEGs with genes that were identified previously in siRNA screens to be important for viral replication [19, 20]. The comparison with a gene list (34 genes) described by Stertz et al. [20] showed little overlap to our DEG gene lists (two to seven genes, data not shown). However, another study [19] used a combination of siRNA experiments and gene expression studies and identified 52 genes. Here, we found an overlap of 18 genes with DEGs from C57BL/6J and 25 with DEGs from DBA/2J (Table 2). Eighteen genes were common to both C57BL/6J and DBA/2J (Table 2, Fig. 4).

**Table 1:** DEG genes

Comparison	DEG-up	DEG-down	DEG-total
B6d1_B6md1	0	0	0
B6d3_B6md3	1012	325	1337
B6d5_B6md3	935	89	1024
B6d8_B6md3	1444	606	2050
B6d14_B6md3	675	362	1037
D2d1_D2md1	0	0	0
D2d3_D2md3	1383	1042	2425
D2d3_D2md5	2246	2289	4535
Comparison	B6-up	D2-up	DEG-total
B6md1_D2md1	32	50	82
B6md3_D2md3	253	225	478
B6d1_D2d1	49	56	105
B6d3_D2d3	84	114	198
B6d5_D2d5	393	119	512

Number of regulated genes from pairwise comparisons. Analysis was performed using LIMMA, setting a threshold of more than 1.4-fold (log_2_ = 0.5) change in expression levels and FDR < 5 %

**Table 2:** DEG genes that overlap genes described in siRNA screens

DEG B6d3	DEG B6d5	DEG D2d3	DEG D2d5	DEG B6D2d3	DEG B6D2d5
AREG	AREG	AREG	AREG	AREG	AREG
ATF3	ATF3	ATF3	ATF3	ATF3	ATF3
B2M	B2M	B2M	B2M	B2M	B2M
BATF2	CASP1	BATF2	BATF2	BATF2	CD274
CD274	CD274	CASP1	CD274	CD274	CXCL2
CXCL2	CXCL2	CD274	CXCL2	CXCL2	IFI44
DUSP5	IFI44	CXCL2	DUSP5	DUSP5	IRF9
FAM46A	IRF9	DUSP5	FKBP11	FAM46A	NFKB2
IFI44	NFKB2	FAM46A	IFI44	IFI44	PHF11
IRF7	PHF11	IFI44	IL15RA	IRF7	STAT1
IRF9	STAT1	IL15RA	IRF7	IRF9	TNFAIP2
LCN2	TNFAIP2	IRF7	IRF9	LCN2	
LGALS3BP	ZC3HAV1	IRF9	LCN2	LGALS3BP	
NFKB2		LCN2	LGALS3BP	NFKB2	
PHF11		LGALS3BP	NFKB2	PHF11	
RNF114		NFKB2	NFKBIA	RNF114	
STAT1		NFKBIA	PHF11	STAT1	
TNFAIP2		PHF11	PPP1R15A	TNFAIP2	
		PLSCR1	RNF114		
		PNPT1	STAT1		
		PPP1R15A	TNFAIP2		
		RNF114			
		STAT1			
		TNFAIP2			
		ZC3HAV1			

List of DEG genes that overlap with genes described by [19]. DEG B6d3, DEG B6d5 genes expressed differentially in infected C57BL/6J compared to mock-treated controls on days 3 and 5 p.i., respectively; DEG D2d3, DEG D2d5 genes expressed differentially in infected DBA/2J compared to mock-treated controls on days 3 and 5 p.i., respectively; DEG B6D2d3, DEG B6D2d5: genes expressed differentially in infected C57BL/6J compared to infected DBA/2J mice on days 3 and 5 p.i., respectively

**Fig. 5:**
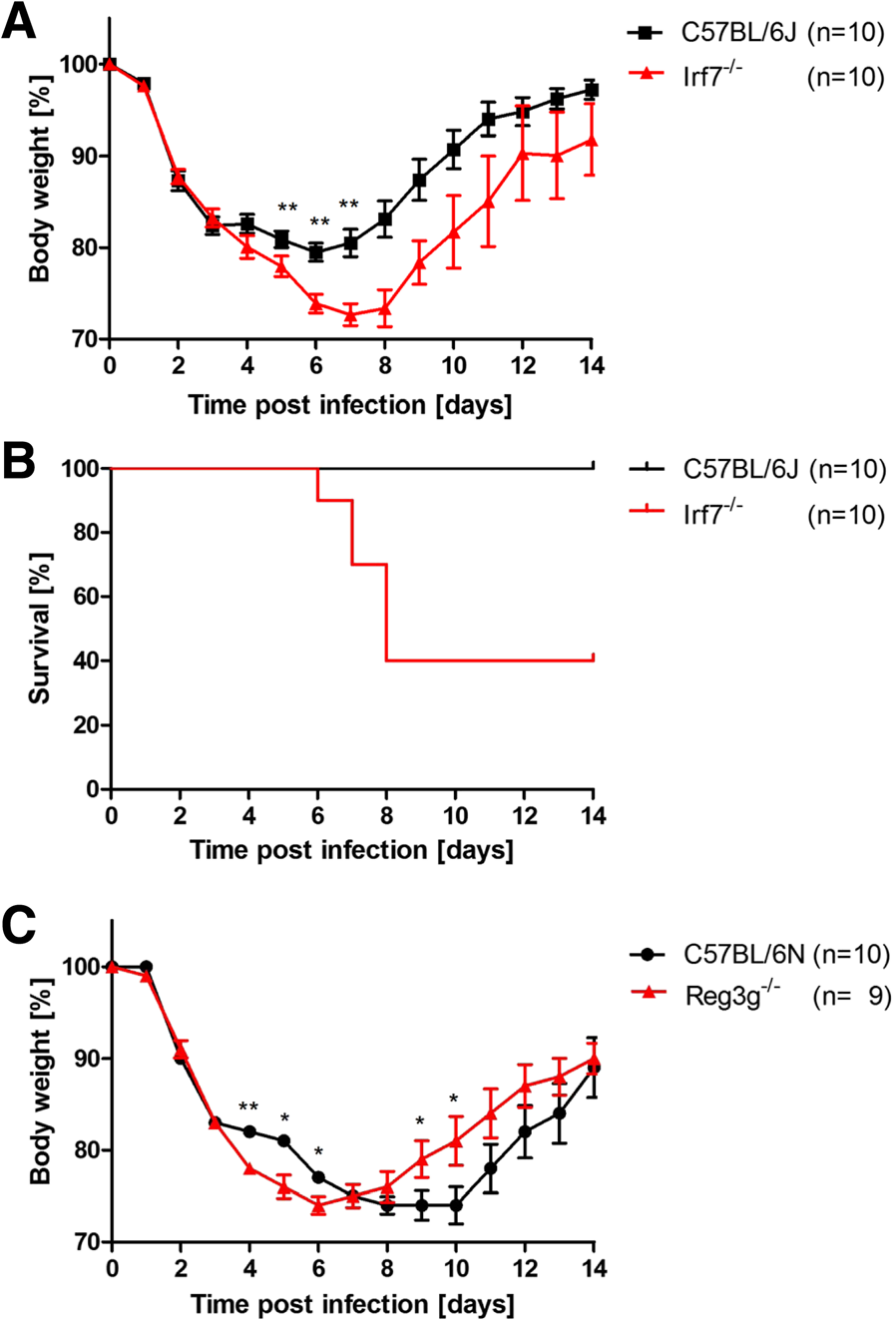
Body weight loss of *Irf7* and *Reg3g* knock-out mice after influenza A infection. Female mice were infected with 2x10^5^ FFU PR8M by intranasal application. Mice with a weight loss of more than 30 % of the starting weight had to be euthanized and were recorded as dead. **a** Homozygous *Irf7* ^*−/−*^ mice showed higher body weight loss (on days 6 to day 8 p.i., *p* < 0.05, Mann Whitney *U* test) and (**b**) significantly increased mortality (Log-rank test, *p* < 0.01) compared to C57BL/6J control mice. **c** Mutant *Reg3g* ^*−/−*^ mice exhibited significant differences in body weight loss at days 4, 5, 6, 9, and 10 p.i. (Mann Whitney *U* test, *p* < 0.05) but no significant increase in mortality (Log-rank test). Please note that in (**a**) after day 6 p.i. only the surviving mice are shown and are thus not representative for the entire group

From these 25 genes that overlapped with DEGs from DBA/2J, we selected *Irf7* (Interferon regulatory factor 7) for further studies. We generated an *Irf7* knock-out line on a C57BL/6J background by backcrossing to test the importance of *Irf7* for the host response to influenza infection. After infection with 2x10^5^ Focus Forming Unit (FFU) PR8M virus, *Irf7*^−/−^ mice lost significantly more body weight and exhibited increased mortality compared to wild type controls (Fig. 5a, b). These observations demonstrate that *Irf7* plays an important role for the host defense to influenza A infection.

### Host genes involved in virus defense and innate immune responses strongly correlate with changes in influenza gene expression

We selected significantly up- or down-regulated genes (FDR < 0.05 and minimal expression level of log_2_ = 1; FDR: false discovery rate) from C57BL/6J infected mice at days 1, 3, 5, 8, and 14 p.i. to identify host genes correlating with the expression of the viral genome. This analysis was restricted to C57BL/6J because we aimed to cover the period of increase in viral load until day 5 p.i. as well as the clearance phase after day 5 p.i. We found 182 host genes with a highly correlated expression (169 positively and 13 negatively) (Spearman correlation coefficient of larger than |0.8|, and FDR < 0.05) (Table 3, Additional file 1: Table S1). Gene Ontology (GO) enrichment analyses of positively correlated genes revealed enrichment for terms including ‘host immune response’, ‘regulation of virus genome replication’, ‘chemokine and cytokine production’ and ‘responses to virus’. Reactome pathway analysis of these 169 positively correlated genes revealed enrichment for terms including ‘interferon signaling’, ‘immune system’, and ‘cytokine signaling in immune system’.

**Table 3:** Host genes for which gene expression levels were highly correlated with influenza gene expression changes in infected C57BL/6J mice

Gene symbol	Coeff	Adj. *p*-value
Ccrl2	0.942857143	0
Tnfaip8l3	0.938338176	2.64E-05
Mst1r	0.921428571	0
Tdrd7	0.921428571	0
2410004I01Rik	0.917857143	0
Fzd5	0.914285714	0
Ifit3	0.914285714	0
Orm1	0.910714286	0
Mx2	0.907142857	0
Dync1h1	0.903571429	0
Serpind1	0.9	0
Cd177	0.892857143	0
Cmpk2	0.892857143	0
I830012O16Rik	0.892857143	0
D14Ertd668e	0.889285714	0
Tnip2	0.889285714	0
Xdh	0.889285714	0
Gp1bb	0.888293474	0.001028469
Asns	0.885714286	0
Flot1	0.882142857	0
Gm13546	0.802502555	0.012013582
Tpmt	−0.814285714	0.012523015
Ift140	−0.829312217	0.006971242
Ano3	−0.82932548	0.006971242
Tmem106c	−0.832142857	0.007760774
Greb1l	−0.835714286	0.006971242
Il7	−0.835714286	0.006971242
Zmat3	−0.835714286	0.006971242
D430041D05Rik	−0.845398014	0.005423668
Atp10b	−0.846428571	0.004637417
Pkhd1	−0.847185325	0.005119055
Cyb5rl	−0.857142857	0.000923657
Sept3	−0.871428571	0
Gpr34	−0.882142857	0

List of top 20 positively correlated genes and all negatively correlated genes (method: Spearman). coeff: Spearman correlation coefficient, adj.p.values: multiple testing corrected FDR. Genes are sorted by decreasing correlation coefficients. The complete list is of all correlating genes is provided in Additional file 1: Table S1

### Several genes are up-regulated in C57BL/6J mice but not in DBA/2J mice after infection

C57BL/6J mice exhibit a much lower viral load in their lungs after infection with H1N1 influenza A virus (PR8M) compared to DBA/2J mice [14, 15]. Furthermore, the host response in DBA/2J is characterized by a stronger inflammatory response [14]. Therefore, we searched for genes that were exclusively up-regulated in C57BL/6J but not in DBA/2J mice after infection with PR8M. We hypothesized that these genes may be responsible for the more efficient control of virus replication in C57BL/6J mice. In a first step, we performed an analysis of variance (ANOVA) for all genes in all groups to identify genes that were significantly up-regulated (FDR < 10 %). From this set, genes up-regulated only in C57BL/6J were selected. This filtering identified five DEGs that were significantly regulated at day 3 and 5 p.i. in C57BL/6J mice: *Lhx2, 2210415F13Rik, Trim15, Reg3g, and Cd72*. The very low expression levels of *Lhx2, 2210415F13Rik and Trim15* make it unlikely that these are crucial candidates mediating the difference in susceptibility between C57BL/6J and DBA/2J. We therefore investigated *Reg3g* (regenerating islet-derived 3 gamma) in more detail.

Knock-out mice carrying a mutation in the *Reg3g* gene on a C57BL/6 N background were infected with influenza PR8M. Differences in body weight loss were observed in mutant compared to wild type mice at day 4 to 6 p.i (Fig. 5c). However, no significant difference in survival was observed between *Reg3g* knock-out and wild type C57BL/6 N mice after influenza A virus infection. Thus, *Reg3g* seems to play a minor role in the host defense to influenza virus H1N1 infection.

## Discussion

Here, we performed RNAseq based analysis of gene expression changes in a murine influenza A infection model by comparing a resistant mouse strain, C57BL/6J, that survives PR8M (H1N1) infection, with a highly susceptible strain, DBA/2J, for which infection with PR8M is lethal. Our studies confirm differences in gene expression profiles between the two mouse strains that were described in a previous analysis using microarrays [18]. At day 3 p.i., 670 differentially expressed probesets in infected C57BL/6J and 1046 in infected DBA/2J mice, respectively, were identified previously by Alberts and colleagues [18] and also overlapped with DEGs found in this study.

Influenza virus transcripts carry a poly(A) tail similar to host mRNAs. Cellular mRNAs are polyadenylated through cleavage at the polyadenylation signal and subsequent addition of the poly(A) tail. In contrast, viral mRNAs obtain their polyadenylation through a stuttering mechanism in which the RNA-dependent RNA polymerase moves back and forth over a stretch of five to seven U residues shortly before the 5´end [21, 22]. Since we selected poly(A) RNAs for RNAseq, we were able to investigate expression of viral genes and, at the same time, to correlate changes in the host transcriptome with increase and decrease of virus gene expression. In this way, we could confirm that changes in expression levels of viral mRNA were correlated with viral load in the infected lungs. The kinetics of viral replication over time as well as the difference between C57BL/6J and DBA/2J was well reflected by changes in sequence counts determined by RNAseq. Thus, the relative changes in RNA expression may serve as a surrogate for virus replication and viral load in infected animals. Thus, RNAseq represents a big advantage compared to microarrays technology where a parallel detection of host gene expression and viral genome expression is not possible.

Zhou et al. [19] combined an siRNA screen with expression analysis in the human lung epithelial cell line A549 after infection with PR8 virus. They identified 300 genes as significantly up-regulated and subsequently performed a siRNA screen for those genes. That screen detected 52 genes as regulators of viral replication, including 40 genes that were not reported previously. We found 25 genes that overlapped with the 52 genes identified by [19] (Table 2).

From the 25 overlapping genes, six genes (*Stat1, B2m, Lgals3bp, Dusp5, Nfkbia, Il15ra*) were also identified as host factors involved in influenza virus replication by Shapira and colleagues [23]. They used human bronchial epithelial cells for transcriptional profiling and combined the data with results from a yeast two-hybrid approach where ten major viral proteins of PR8 were tested against 12,000 human proteins.

Furthermore, genes acting downstream of RIG-I binding to viral RNA like *Irf7, Irf9, Stat1* and *NF-kB* were also found amongst the genes that overlapped with the list from Zhou et al. [19]. Amongst these factors, IRF7 has been described as an essential key mediator of interferon signaling activation and regulation and has been shown to be critical for innate immunity [24]. It is constitutively expressed in plasmacytoid dendritic cells which rapidly produce type I IFN in response to viral infection [25, 26]. This initial activation triggers a positive feedback loop regulation of *Ifnα* and *Ifnβ* genes by *Irf7* in adjacent cells [27, 28]. The importance of *Irf7* in influenza pathogenesis was also shown in several *in vitro* studies [29, 30]. Epithelial cells recognize influenza A virus via RIG-I/MAVS, leading to the activation of *Irf7* and subsequent induction of type I and type III interferons in redundant amplification loops. In addition, a recently published study revealed an IRF7-dependent amplification of IFNs in an influenza patient carrying a mutation in that gene [31]. In contrast, no *in vivo* studies using *Irf7* deficient mouse mutants have been published so far. Therefore, we selected *Irf7* (Interferon regulatory factor 7) to generate knock-out mice on a C57BL/6J background by backcrossing and to investigate its role for host defense *in vivo.* After infection with PR8M, *Irf7*-deficient mice exhibited a more pronounced body weight loss and increased mortality compared to wild type mice after infection with H1N1 virus. These experiments demonstrate the *in vivo* relevance of *Irf7* for the host response to influenza virus infection. Our studies confirm the potential role of *Irf7* in influenza pathogenesis in an *in vivo* model system as suggested by previous *in vitro* studies [29, 30]. The potential functional roles of all other genes from the list in Table 2 are discussed in more detail in the supplements.

When comparing results from several RNAi screens [23, 32–35], Stertz and Shaw identified 34 genes with potential importance for viral replication that were found in at least two screens (reviewed in [20]). However, only seven genes overlapped with the 34 genes in DBA/2J at day 5 p.i. (*Plk3, Rps10, Il17ra, Ptprn, Racgap1, Nhp2l1, Atp6v0c*). One explanation for the small overlap may be that the RNAi screens were performed in cell culture whereas our studies identified differentially regulated genes in infected lungs. It should be noted that transcriptomes in lungs are much more complex due to the contribution from infiltrating immune cells. Thus, changes in expression of cultured cells may not reflect the entire spectrum of host responses well. More future studies will be necessary to further elucidate this aspect.

Since viral and host transcripts can be followed in the same individual, we were able to correlate changes in host gene expression with changes in the level of virus gene expression. We studied host gene expression in C57BL/6J lungs and viral transcripts including both the period of increasing viral load (day 1 to day 5 p.i.) as well as the period of decrease in viral load (day 8 to 14 p.i.). We found 182 host genes that were positively or negatively correlated with influenza gene expression in infected C57BL/6J mice (Table 3 shows the 20 positively correlated genes and all negatively correlated genes). Many of these genes exhibit well known functions in the host immune response which are discussed in more detail in the Additional file 2: Supplemental Material.

In contrast to C57BL/6J mice that survive, DBA/2J mice die on day six to seven after infection with PR8. A comparison of the DEGs in both mouse strains was performed to identify genes that are exclusively up-regulated in C57BL/6J. We hypothesize that these genes are candidates mediating the resistance of C57BL/6J against influenza infection. We identified two genes (*Cd72 and Reg3g*) that were significantly and strongly up-regulated in C57BL/6J mice compared to DBA/2J. The B cell co-receptor Cd72 is an important receptor regulating B cell activation [36], negatively regulating BCR signaling [37] and is additionally expressed on murine NK cells where it acts in an inhibitory manner through regulating cytokine production but not cytotoxicity [38]. In C57BL/6J we observed a two-fold higher up-regulation of Cd72 compared to DBA/2J. The resulting deficit in the inhibitory effect on NK cells and the following diminished regulation of cytokine amounts may be a good explanation for the exaggerated immune response observed in DBA/2J mice. More experiments will be needed to evaluate the possible role of Cd72 for the host response to influenza A virus.

For *Reg3g* an increase in expression levels was observed in IBD (inflammatory bowel disease), a murine bacterial reconstitution model [39] and after experimental intestinal infection with *Listeria monocytogenes* [40]. The role of *Reg3g* in lung infection was further elucidated by Choi et al. [41]. They were able to show that *Reg3g* expression is regulated by Stat3 and highly increased after MRSA (Methicillin-resistant *Staphylococcus aureus*) infection in the lung epithelium. Administration of recombinant *Reg3g* was able to restore mucosal immunity against MRSA *in vivo*, highlighting the therapeutic potential for *Reg3g* [41]. The fact that *Reg3g* was up-regulated in C57BL/6J, but not in DBA/2J may account for the differences in disease outcome. We therefore studied the possible role of *Reg3g* in knock-out mice. However, despite its strong up-regulation after influenza virus infection, deletion of this gene had no strong effect on the susceptibility of the host to infections with H1N1. It may, however, be possible that *Reg3g* deficient mice are susceptible to other influenza virus subtypes or other viral infections.

## Conclusions

In conclusion, using RNAseq analysis we identified novel genes important for viral replication or host defense. This study adds further important knowledge to host-pathogen-interactions and suggests additional candidates that are crucial for host susceptibility or survival during influenza A infections.

## Methods

### Ethics statement

All experiments in mice were approved by an external committee according to the German national guidelines of the animal welfare law. The protocol used in these experiments has been reviewed and approved by an ethics committee as described in the regulations from the German Bundesministerium für Ernährung, Landwirtschaft und Verbraucherschutzand, and detailed in the “Tierschutzkommissions-Verordnung vom 23. Juni 1987 (BGBl. I S. 1557)” (http://www.gesetze-im-internet.de/bundesrecht/tierschkomv/gesamt.pdf). Subsequently, the protocol has been formally approved by the ‘Niedersächsisches Landesamt für Verbraucherschutz und Lebensmittelsicherheit, Oldenburg, Germany’ (Permit Number: 3392 42502-04-13/1234).

### Virus and mice

The mouse-adapted virus strain influenza A/Puerto Rico/8/1934 H1N1 (PR8M) was produced as described previously [14, 42]. C57BL/6J and DBA/2J mice were obtained from Janvier, France. Mutant B6;129P2-*Irf7*^tm1Ttg^ were kindly provided by Tadatsugu Taniguchi [24]. B6;129P2-*Irf7*^tm1Ttg^ mice were backcrossed to C57BL/6J for 12 generations to generate B6.129P2-*Irf7*^tm1Ttg^ mice (*Irf7*^*−/−*^). The background was confirmed by SNP-genotyping (Mouse Universal Genotyping Array (MUGA), Neogen Corporation, USA). The *Reg3g* knock-out mouse strain was created from ES cell clone EPD0309_D08, obtained from the KOMP Repository (www.komp.org) to generate B6-*Reg3g*^tm1a(KOMP)Wtsi^ (*Reg3g*^−/−^) mice.

### Mouse infections

Female, 10–12 weeks old mice were anesthetized by intra-peritoneal injection with Ketamine/Xylazine (85 % NaCl (0.9 %), 10 % Ketamine, 5 % Xylazine) with doses adjusted to the individual body weight. Mice were then intranasally infected with 20 μl virus solution (2x10^3^ (RNASeq) or 2x10^5^ (knock-out mice) FFU PR8M) or mock-infected with PBS.

### RNA isolation

Mice were sacrificed and entire lungs were extracted from mice from both strains on days 1, 3 and 5 after infection. For mock-infected animals, mice were sacrificed at days 1 and 3 post treatment. In addition, lungs from C57BL/6J mice were also collected on days 8 and 14. For every treatment and day post infection (p.i.) 4–5 mice were prepared. The lungs were immediately transferred to RNAlater solution (Qiagen), kept at 4 °C for one day and subsequently stored at −20 °C. RNA was isolated using Qiagen Midi Kit as described previously [43]. RNA quality was controlled on a 2100 Bioanalyzer Instrument (Agilent). All RNA samples had a RNA Integrity Number (RIN) of ≥ 9.7. Three independent biological replicates were selected for each time point for subsequent RNA sequencing.

### RNAseq library preparation, sequencing and analysis

Twenty μg of total RNA was enriched for poly A+ RNA using one cycle of the Poly A Purist Kit from Ambion according to the manufacturer’s standard protocol. The resulting enriched RNA samples were analyzed on an Agilent Bioanalyzer to determine the remaining amount of rRNA in the samples. If the amount was higher than 5 %, samples were subjected to another cycle of Poly A enrichment. One-hundred ng of the poly A+ enriched RNA was then used to prepare libraries for sequencing using the AB Library Builder™ Whole Transcriptome Core Kit for 5500 Genetic Analysis Systems on a Library Builder system. Libraries were amplified for 15 cycles before 5500 Wildfire primers were added using five cycles of fusion primer amplification as directed in the 5500 Wildfire manual. Before sequencing, small aliquots of libraries were pooled and sequenced on an Ion Torrent PGM 314 chip after additional amplification with PGM fusion primers. The library pools were quantified by Real-Time PCR and immobilized on flow cells for the SOLiD 5500 Wildfire instrument (Applied Biosystems) and sequenced (50 bp reads). The average number of reads per sample was 29.5. One sample had a high number of reads (230 million), and the others on average had 23 million reads. The mouse reference genome (GRCm38/mm10) was downloaded from ftp://hgdownload.cse.ucsc.edu/goldenPath/mm10/chromosomes/. Sequencing reads (XSQ format) from C57BL/6J samples were aligned to the C57BL/6J reference genome using the Whole Transcriptome mapping module of the LifeScope 2.5.1 software (http://www.lifetechnologies.com/lifescope). Similarly, sequencing reads from DBA/2J samples were aligned to the enhanced DBA/2J genome that was generated by substituting ~4.5 million DBA/2J SNPs in the reference genome. Filter reference containing polyA, polyC, polyG, polyT, rRNAs, tRNAs, as well as adaptor, barcode, and primer sequences was used to remove non-mRNAs reads prior to the mapping. We used the mouse RefSeq transcript annotation downloaded from UCSC genome browser (www.genome.ucsc.edu) to generate a junction reference library containing a list of exon-exon pairs. Reads were aligned against both the reference genome and the junction library. Reads that could not be mapped were realigned against the H1N1 viral contigs. Reads with minimum mapping quality of 10 were used to generate raw counts to be used for downstream differential and correlation analysis.

### Bioinformatic analysis

Raw read counts were used for analysis with DESeq2 [44] statistical package after adding 1 to all values. The DESeq function rlogTransformation was used to normalize and log transform raw read counts and to calculate normalized expression counts. The normalized expression counts were then used for further analysis without applying any additional pre-processing filtering. Principal component analysis analysis and identification of differentially expressed genes were performed using DESeq2. DEGs were selected based on an adjusted *p*-value of 0.05 (FDR of 5 %) and exhibiting at least a 1.4-fold difference in expression levels (log_2_ = 0.5). Strip charts, scatter plots and heat maps were generated using the R software package [45]. Multi-group comparisons were performed with the LIMMA package [46] using BH correction for multiple testing [47]. Cell signature genes were identified based on the BioGPS database (GEO database ID GSE10246) and our previous analysis of gene expression patterns in a non-lethal infection [17]. Inflammatory genes that are expressed during influenza infections were selected based on our previous influenza transcriptome studies [17, 43]. For analysis of influenza transcripts, log_2_-transformed RPKM values were calculated from counts of sequences that aligned to influenza gene segments. Analysis of correlations between influenza (log_2_ RPKM counts of the sum of all genes) and host gene (normalized log_2_ counts) expression levels was performed with the R function cor using Spearman as method. Correlation graphs were generated using the R package ‘corrgram’ [48]. Adjusted *p*-values for correlated genes were calculated as FDR using cor.test. GO enrichment analysis and Reactome enrichment analysis (using the full gene list from normalized counts as reference) was performed with the R package clusterProfiler [49].

### Availability of supporting data

The raw RNAseq data has been deposited at GEO (http://www.ncbi.nlm.nih.gov/geo/) under accession number GSE66040 (http://www.ncbi.nlm.nih.gov/geo/query/acc.cgi?acc=GSE66040).

## Additional files

Additional file 1: Table S1. Genes expressed in infected C57BL/6J at days 1, 3, 5, 8, and 14 p.i. that strongly correlate with changes in expression of influenza segments. (PDF 76 kb) Additional file 2:
**Supplemental Material.** Detailed discussion of known biological functions of genes from Tables 2 and 3. (PDF 65 kb)
