# Supplementary material for: RNAseq expression analysis of resistant and susceptible mice after influenza A virus infection identifies novel genes associated with virus replication and important for host resistance to infection
Source: BMC Genomics. 2015 Sep 2;16(1):655. doi: 10.1186/s12864-015-1867-8 (PMC4557482; doi:10.1186/s12864-015-1867-8)
Supplement: Additional file 1: Table S1. — Genes expressed in infected C57BL/6J at days 1, 3, 5, 8, and 14 p.i. that strongly correlate with changes in expression of influenza segments. (PDF 76 kb) [file 12864_2015_1867_MOESM1_ESM.pdf]

Table S1
Complete list of all correlating genes

gene symbol	coeff	FDR
Ccrl2	0,942857143	0
Tnfaip8l3	0,938338176	2,64E-05
Mst1r	0,921428571	0
Tdrd7	0,921428571	0
2410004I01Rik	0,917857143	0
Fzd5	0,914285714	0
Ifit3	0,914285714	0
Orm1	0,910714286	0
Mx2	0,907142857	0
Dync1h1	0,903571429	0
Serpind1	0,9	0
Cd177	0,892857143	0
Cmpk2	0,892857143	0
I830012O16Rik	0,892857143	0
D14Ertd668e	0,889285714	0
Tnip2	0,889285714	0
Xdh	0,889285714	0
Gp1bb	0,888293474	0,001028469
Asns	0,885714286	0
Flot1	0,882142857	0
Itih4	0,882142857	0
Uap1	0,882142857	0
Ccl11	0,88114423	0,001458024
Rhcg	0,88114423	0,001458024
Ggt5	0,878571429	0
AW011738	0,875	0
H6pd	0,875	0
Papd7	0,875	0
2310014L17Rik	0,871428571	0
Lrrc8a	0,871428571	0
Maff	0,871428571	0
Ms4a4d	0,871428571	0
Taf7	0,871428571	0
Tpst1	0,871428571	0
Csf2	0,867857143	0
Cxcl14	0,867857143	0
Gadd45b	0,867857143	0
Ifit2	0,867857143	0
Ldlr	0,867857143	0
Map3k6	0,867857143	0
Ubr4	0,867857143	0
Gch1	0,865833228	0,002831401
2010002M12Rik	0,864285714	0
Il1f9	0,864285714	0
Isg15	0,864285714	0

Lrp1	0,864285714	0
Sele	0,864285714	0
Serpina3m	0,864285714	0
Usp18	0,864285714	0
Ddit3	0,860714286	0
F7	0,860714286	0
Pmvk	0,860714286	0
Gdap10	0,859696501	0,003499373
Nt5c3	0,859696501	0,003499373
Ifit1	0,857142857	0,000923657
Naa20	0,857142857	0,000923657
Fgg	0,856121879	0,003903554
Ampd3	0,853571429	0,002276947
Parp12	0,853571429	0,002276947
Parp14	0,853571429	0,002276947
Pttg1	0,853571429	0,002276947
Duoxa2	0,853052074	0,004378705
AA986860	0,852547257	0,004407812
Anxa7	0,85	0,003499373
Chi3l1	0,85	0,003499373
Gm14446	0,85	0,003499373
Gsn	0,85	0,003499373
1190002H23Rik	0,846428571	0,004637417
Ern1	0,846428571	0,004637417
Stat2	0,846428571	0,004637417
Ifih1	0,842857143	0,005779315
Prkx	0,842857143	0,005779315
Pydc3	0,842857143	0,005779315
Sphk1	0,842857143	0,005779315
Ddx4	0,841823392	0,005779315
Tor1aip1	0,841823392	0,005779315
Dlgap4	0,839285714	0,006623604
Hspa5	0,839285714	0,006623604
Hspb1	0,839285714	0,006623604
Ifi35	0,839285714	0,006623604
Ovol1	0,839285714	0,006623604
Slfn5	0,839285714	0,006623604
Whamm	0,839285714	0,006623604
Ptcd1	0,838248771	0,006542561
Asb13	0,835714286	0,006971242
Ascc3	0,835714286	0,006971242
Clcn6	0,835714286	0,006971242
Ctla2a	0,835714286	0,006971242
Fam46b	0,835714286	0,006971242
Oasl1	0,835714286	0,006971242
Smtnl2	0,835714286	0,006971242
Trim26	0,835714286	0,006971242
Orm2	0,834674149	0,006816087
2210020M01Rik	0,833632819	0,006971242
2900064A13Rik	0,832142857	0,007760774

Herc6	0,832142857	0,007760774
Ifitm2	0,832142857	0,007760774
Lipg	0,832142857	0,007760774
Rhoj	0,832142857	0,007760774
2210415F13Rik	0,831843907	0,006971242
Cxcl1	0,831099528	0,006971242
Oas1b	0,831099528	0,006971242
4930599N23Rik	0,828571429	0,0086098
Ankfy1	0,828571429	0,0086098
Lysmd2	0,828571429	0,0086098
Scarb2	0,828571429	0,0086098
Xaf1	0,828571429	0,0086098
A630012P03Rik	0,827524906	0,007357479
Atf5	0,825	0,009742302
H2-K2	0,825	0,009742302
Hspa1b	0,825	0,009742302
Prss23	0,825	0,009742302
Gal	0,824688261	0,007760774
Zufsp	0,823950284	0,007760774
Rrs1	0,822162974	0,008179654
Adcy4	0,821428571	0,010449739
Dll1	0,821428571	0,010449739
Esd	0,821428571	0,010449739
Fdps	0,821428571	0,010449739
Ifi44	0,821428571	0,010449739
Ifitm3	0,821428571	0,010449739
Map2k1	0,821428571	0,010449739
Nod1	0,821428571	0,010449739
Fga	0,821110437	0,008405364
Chac1	0,820375663	0,008544272
March5	0,817857143	0,01131956
Anxa3	0,817857143	0,01131956
Egfr	0,817857143	0,01131956
Mob3c	0,817857143	0,01131956
Mx1	0,817857143	0,01131956
Sap30	0,817857143	0,01131956
Tinagl1	0,817857143	0,01131956
Znfx1	0,817857143	0,01131956
Masp1	0,816801041	0,009164885
Dhrs9	0,814285714	0,012523015
Fkbp1a	0,814285714	0,012523015
Rnf121	0,814285714	0,012523015
Best1	0,813226419	0,009903452
Sct	0,812165879	0,010169103
Hpx	0,811439109	0,010329847
C2	0,810714286	0,0135206
Chmp4b	0,810714286	0,0135206
Mxd1	0,810714286	0,0135206
Npc2	0,810714286	0,0135206
Psma7	0,810714286	0,0135206

Samd9l	0,810714286	0,0135206
Trim16	0,810714286	0,0135206
Adamtsl1	0,807142857	0,013899478
Adck4	0,807142857	0,013899478
Apod	0,807142857	0,013899478
Fth1	0,807142857	0,013899478
Hmgcs1	0,807142857	0,013899478
Ifi27l2b	0,807142857	0,013899478
Irf9	0,807142857	0,013899478
Mreg	0,807142857	0,013899478
Nmi	0,807142857	0,013899478
Pcgf5	0,807142857	0,013899478
S100a9	0,807142857	0,013899478
Saa3	0,807142857	0,013899478
Spon1	0,807142857	0,013899478
Trim25	0,807142857	0,013899478
Snhg1	0,806077176	0,01131956
Cebpb	0,803571429	0,015014999
Gadd45g	0,803571429	0,015014999
Sowahc	0,803571429	0,015014999
Steap4	0,803571429	0,015014999
Tox2	0,803571429	0,015014999
Wdfy1	0,803571429	0,015014999
Gm13546	0,802502555	0,012013582
Tpmt	-0,814285714	0,012523015
Ift140	-0,829312217	0,006971242
Ano3	-0,82932548	0,006971242
Tmem106c	-0,832142857	0,007760774
Greb1l	-0,835714286	0,006971242
Il7	-0,835714286	0,006971242
Zmat3	-0,835714286	0,006971242
D430041D05Rik	-0,845398014	0,005423668
Atp10b	-0,846428571	0,004637417
Pkhd1	-0,847185325	0,005119055
Cyb5rl	-0,857142857	0,000923657
Sept3	-0,871428571	0
Gpr34	-0,882142857	0
