# Supplementary material for: RNAseq expression analysis of resistant and susceptible mice after influenza A virus infection identifies novel genes associated with virus replication and important for host resistance to infection
Source: BMC Genomics. 2015 Sep 2;16(1):655. doi: 10.1186/s12864-015-1867-8 (PMC4557482; doi:10.1186/s12864-015-1867-8)
Supplement: Additional file 2: — Supplemental Material. Detailed discussion of known biological functions of genes from Tables 2 and 3. (PDF 65 kb) [file 12864_2015_1867_MOESM2_ESM.pdf]

## Supplemental materials

### Detailed discussion of 25 overlapping genes (Table 2)

Zhou et al [1] combined an siRNA screen with expression analysis in the human lung epithelial cell line A549 after infection with PR8 virus. They identified 300 genes as significantly up-regulated and subsequently performed an siRNA screen for these genes. This screen detected 52 genes as regulators of viral replication, including 40 genes that were not reported previously. We found 25 genes that overlapped with the 52 genes identified by [1].

One of these genes, *Fmr1*, was investigated by others in detail and its relevance for viral ribonucleoprotein assembly could be demonstrated *in vitro* and *in vivo* [1].

From the 25 overlapping genes, six genes (*Stat1, B2m, Lgals3bp, Dusp5, Nfkbia, Il15ra*) were also identified as host factors involved in influenza virus replication by Shapira and colleagues [2]. They used human bronchial epithelial cells for transcriptional profiling and combined the data with results from a yeast two-hybrid approach where ten major viral proteins of PR8 were tested against 12,000 human proteins.

Furthermore, genes acting downstream of RIG-I binding to viral RNA like *Irf7, Irf9, Stat1* and *NF-kB* were also found amongst the genes that overlapped with the list from Zhou et al. [1]. STAT1, IRF9 and STAT2 form a heterotrimer, known as the ISGF3 complex that mediates induction of interferon stimulated genes. Another candidate with reported antiviral activity, *ZC3HAV1* (zinc finger CCCH type, antiviral 1) correlated with survival of chicken infected with H5N1 variants [3]. *Plscr1* is induced by interferon and is involved in amplifying the interferon response [4]. It is a TLR9-interacting protein that plays an important role in pDCs type 1 IFN responses by regulating TLR9 trafficking to the endosomal compartment [5]. We speculate that genes like *Cxcl2, Lcn2, Il15ra, LgalS3BP, Casp1, Cd274, Tnfaip2 and Atf3 (a negative regulatory transcription factor in TLR pathways)* reflect the pathogen induced activation of macrophages. The detrimental role of *Cd274* (programmed death ligand 1) in influenza infection was demonstrated recently [6-8]. The knockout of caspase 1 (*Casp1*), a key mediator of inflammatory processes revealed a higher susceptibility to influenza [9, 10]. The importance of the transcription factor NF-kB for viral replication has also been shown [11, 12]. Recently this pathway was assessed as potential therapeutic target [13].

**Detailed discussion of correlating genes (Table 3)**

Since viral and host transcripts can be followed in the same individual, we were able to correlate changes in host gene expression with changes in the level of virus gene expression. We studied host gene expression in C57BL/6J lungs and viral transcripts including both the period of increasing viral load (day 1 to day 5 p.i.) as well as the period of decrease in viral load (day 8 to 14 p.i.). We found 182 host genes that were positively or negatively correlated with influenza gene expression in infected C57BL/6J mice.

The role in immune response processes of many of the positively correlated genes is already known. For example, it has been shown that *Flotillin 1* (*Flot1*) plays an important role during neutrophil recruitment and migration [14] whereas *Orm1* inhibits neutrophil migration to the focus of infection after polymicrobial sepsis [15].

Porcine *Ifit3* is highly induced by IFN-α/β and swine influenza virus (SIV) and is able to inhibit SIV replication as well as to enhance IFN-β production [16]. Moreover increased expression of *Ifit3* has been revealed during lymphocytic choriomeningitis virus (LCMV) and West Nile virus (WNV) infection in the murine central nervous system (CNS). Thus, a dominant role in the host response to different viruses in the CNS was suggested [17].

Other genes interfere with pathways known to play important roles in the immune response after influenza A virus infection: *Tnip2* which is required for optimal activation of the Erk signaling pathway and the inhibition of NfκB activation [18-21]. It has been shown recently that the macrophage stimulating 1 receptor (*Mst1r*) plays an important role in controlling cytokine secretion in inflammatory bowel disease (IBD) [22], negatively regulates TNFα production in alveolar macrophages [23] and its signaling promotes cell survival [24]. Additionally, the tyrosine kinase of this receptor plays a regulatory role in the immune response during acute lung injury [25]. The function of several of these genes has already been validated in knock-out mouse models: *Ccrl2* deficient mice exhibit alteration in trafficking of antigen-loaded lung dendritic cells and the authors suggest that this receptor might control excessive airway inflammatory responses [26]. In *Serpind1* KO mice an increased susceptibility to *Pseudomonas aeruginosa* was observed and a new role of *Serpind1* as a factor of host defense in innate immunity was concluded [27].

Among the negatively correlated genes, *interleukin-7* (*Il7*) represents a critical cytokine for the induction of T follicular helper (Tfh) cells [28]. The negative correlation

may account for the fact that the immune response can have beneficial as well as detrimental effects to the host. Genes that are highly expressed and are negatively correlated with flu counts are: *Ift140*, *Tmem106c*, *Zmat3*, *Pkhd1* and *Cyb5rl.*
Several genes that overlap with previously identified siRNA genes, such as *Dusp5, B2m, Pnpt1, Areg, Fam46a, Ppp1r15a, Tnfaip2,* have not yet been associated with the host response to influenza virus infections. Similarly, for several genes that were positively correlated with virus genome expression and expressed at high levels in the lung after influenza infection, no biological function has been described yet: *Tdrd7*, *Dync1h1*, *CD177*, *I830012O16Rik* and *D14Ertd668e.* These genes are promising candidates for further investigations.

## References


1. Zhou Z, Cao M, Guo Y, Zhao L, Wang J, Jia X, Li J, Wang C, Gabriel G, Xue Q *et al*: **Fragile X mental retardation protein stimulates ribonucleoprotein assembly of influenza A virus**. *Nat Commun* 2014, **5**:3259.
2. Shapira SD, Gat-Viks I, Shum BO, Dricot A, de Grace MM, Wu L, Gupta PB, Hao T, Silver SJ, Root DE *et al*: **A physical and regulatory map of host-influenza interactions reveals pathways in H1N1 infection**. *Cell* 2009, **139**(7):1255-1267.
3. Uchida Y, Watanabe C, Takemae N, Hayashi T, Oka T, Ito T, Saito T: **Identification of host genes linked with the survivability of chickens infected with recombinant viruses possessing H5N1 surface antigens from a highly pathogenic avian influenza virus**. *J Virol* 2012, **86**(5):2686-2695.
4. Dong B, Zhou Q, Zhao J, Zhou A, Harty RN, Bose S, Banerjee A, Slee R, Guenther J, Williams BR *et al*: **Phospholipid scramblase 1 potentiates the antiviral activity of interferon**. *J Virol* 2004, **78**(17):8983-8993.
5. Talukder AH, Bao M, Kim TW, Facchinetti V, Hanabuchi S, Bover L, Zal T, Liu YJ: **Phospholipid scramblase 1 regulates Toll-like receptor 9-mediated type I interferon production in plasmacytoid dendritic cells**. *Cell Res* 2012, **22**(7):1129-1139.
6. Valero-Pacheco N, Arriaga-Pizano L, Ferat-Osorio E, Mora-Velandia LM, Pastelin-Palacios R, Villasis-Keever MA, Alpuche-Aranda C, Sanchez-Torres LE, Isibasi A, Bonifaz L *et al*: **PD-L1 expression induced by the 2009**

**pandemic influenza A(H1N1) virus impairs the human T cell response**. *Clin Dev Immunol* 2013, **2013**:989673.

7. McNally B, Ye F, Willette M, Flano E: **Local blockade of epithelial PDL-1 in the airways enhances T cell function and viral clearance during influenza virus infection**. *J Virol* 2013, **87**(23):12916-12924.
8. Maazi H, Singh AK, Speak AO, Lombardi V, Lam J, Khoo B, Inn KS, Sharpe AH, Jung JU, Akbari O: **Lack of PD-L1 expression by iNKT cells improves the course of influenza A infection**. *PLoS ONE* 2013, **8**(3):e59599.
9. Huang CH, Chen CJ, Yen CT, Yu CP, Huang PN, Kuo RL, Lin SJ, Chang CK, Shih SR: **Caspase-1 deficient mice are more susceptible to influenza A virus infection with PA variation**. *J Infect Dis* 2013, **208**(11):1898-1905.
10. Thomas PG, Dash P, Aldridge JR, Jr., Ellebedy AH, Reynolds C, Funk AJ, Martin WJ, Lamkanfi M, Webby RJ, Boyd KL *et al*: **The intracellular sensor NLRP3 mediates key innate and healing responses to influenza A virus via the regulation of caspase-1**. *Immunity* 2009, **30**(4):566-575.
11. Wurzer WJ, Ehrhardt C, Pleschka S, Berberich-Siebelt F, Wolff T, Walczak H, Planz O, Ludwig S: **NF-kappaB-dependent induction of tumor necrosis factor-related apoptosis-inducing ligand (TRAIL) and Fas/FasL is crucial for efficient influenza virus propagation**. *J Biol Chem* 2004, **279**(30):30931-30937.
12. Ruckle A, Haasbach E, Julkunen I, Planz O, Ehrhardt C, Ludwig S: **The NS1 protein of influenza A virus blocks RIG-I-mediated activation of the noncanonical NF-kappaB pathway and p52/RelB-dependent gene expression in lung epithelial cells**. *J Virol* 2012, **86**(18):10211-10217.
13. Haasbach E, Reiling SJ, Ehrhardt C, Droebner K, Ruckle A, Hrincius ER, Leban J, Strobl S, Vitt D, Ludwig S *et al*: **The NF-kappaB inhibitor SC75741 protects mice against highly pathogenic avian influenza A virus**. *Antiviral Res* 2013.
14. Ludwig A, Otto GP, Riento K, Hams E, Fallon PG, Nichols BJ: **Flotillin microdomains interact with the cortical cytoskeleton to control uropod formation and neutrophil recruitment**. *J Cell Biol* 2010, **191**(4):771-781.
15. Spiller F, Carlos D, Souto FO, de Freitas A, Soares FS, Vieira SM, Paula FJ, Alves-Filho JC, Cunha FQ: **alpha1-Acid glycoprotein decreases neutrophil**

**migration and increases susceptibility to sepsis in diabetic mice**. *Diabetes* 2012, **61**(6):1584-1591.

16. Li Y, Wen Z, Zhou H, Wu S, Jia G, Wei Q, Jin M: **Porcine interferon-induced protein with tetratricopeptide repeats 3, poIFIT3, inhibits swine influenza virus replication and potentiates IFN-beta production**. *Dev Comp Immunol* 2014.
17. Wacher C, Muller M, Hofer MJ, Getts DR, Zabaras R, Ousman SS, Terenzi F, Sen GC, King NJ, Campbell IL: **Coordinated regulation and widespread cellular expression of interferon-stimulated genes (ISG) ISG-49, ISG-54, and ISG-56 in the central nervous system after infection with distinct viruses**. *J Virol* 2007, **81**(2):860-871.
18. Leotoing L, Chereau F, Baron S, Hube F, Valencia HJ, Bordereaux D, Demmers JA, Strouboulis J, Baud V: **A20-binding inhibitor of nuclear factor-kappaB (NF-kappaB)-2 (ABIN-2) is an activator of inhibitor of NF-kappaB (IkappaB) kinase alpha (IKKalpha)-mediated NF-kappaB transcriptional activity**. *J Biol Chem* 2011, **286**(37):32277-32288.
19. Papoutsopoulou S, Symons A, Tharmalingham T, Belich MP, Kaiser F, Kioussis D, O'Garra A, Tybulewicz V, Ley SC: **ABIN-2 is required for optimal activation of Erk MAP kinase in innate immune responses**. *Nat Immunol* 2006, **7**(6):606-615.
20. Li CC, Chou CK, Wang MH, Tsai TF: **Overexpression of ABIN-2, a negative regulator of NF-kappaB, delays liver regeneration in the ABIN-2 transgenic mice**. *Biochem Biophys Res Commun* 2006, **342**(1):300-309.
21. Van Huffel S, Delaei F, Heyninck K, De Valck D, Beyaert R: **Identification of a novel A20-binding inhibitor of nuclear factor-kappa B activation termed ABIN-2**. *J Biol Chem* 2001, **276**(32):30216-30223.
22. Kulkarni RM, Stuart WD, Gurusamy D, Waltz SE: **Ron receptor signaling is protective against DSS-induced colitis in mice**. *Am J Physiol Gastrointest Liver Physiol* 2014, **306**(12):G1065-1074.
23. Nikolaidis NM, Kulkarni RM, Gray JK, Collins MH, Waltz SE: **Ron receptor deficient alveolar myeloid cells exacerbate LPS-induced acute lung injury in the murine lung**. *Innate immunity* 2011, **17**(6):499-507.
24. Logan-Collins J, Thomas RM, Yu P, Jaquish D, Mose E, French R, Stuart W, McClaine R, Aronow B, Hoffman RM *et al*: **Silencing of RON receptor**

**signaling promotes apoptosis and gemcitabine sensitivity in pancreatic cancers**. *Cancer Res* 2010, **70**(3):1130-1140.

25. Nikolaidis NM, Gray JK, Gurusamy D, Fox W, Stuart WD, Huber N, Waltz SE: **Ron receptor tyrosine kinase negatively regulates TNFalpha production in alveolar macrophages by inhibiting NF-kappaB activity and Adam17 production**. *Shock* 2010, **33**(2):197-204.
26. Otero K, Vecchi A, Hirsch E, Kearley J, Vermi W, Del Prete A, Gonzalvo-Feo S, Garlanda C, Azzolino O, Salogni L *et al*: **Nonredundant role of CCRL2 in lung dendritic cell trafficking**. *Blood* 2010, **116**(16):2942-2949.
27. Kalle M, Papareddy P, Kasetty G, Tollefsen DM, Malmsten M, Morgelin M, Schmidtchen A: **Proteolytic activation transforms heparin cofactor II into a host defense molecule**. *J Immunol* 2013, **190**(12):6303-6310.
28. Seo YB, Im SJ, Namkoong H, Kim SW, Choi YW, Kang MC, Lim HS, Jin HT, Yang SH, Cho ML *et al*: **Crucial roles of interleukin-7 in the development of T follicular helper cells and in the induction of humoral immunity**. *J Virol* 2014, **88**(16):8998-9009.
